# Atezolizumab Treatment for Progressive Multifocal Leukoencephalopathy

**DOI:** 10.3201/eid2801.204809

**Published:** 2022-01

**Authors:** Nicolas Lambert, Solène Dauby, Dominique Dive, Bernard Sadzot, Pierre Maquet

**Affiliations:** University Hospital of Liège, Liège, Belgium

**Keywords:** progressive multifocal leukoencephalopathy, immunotherapy, JC virus, immune reconstitution inflammatory syndrome, Belgium, viruses, atezolizumab

## Abstract

Atezolizumab successfully reinvigorated JC virus immunity in a patient in Belgium with progressive multifocal leukoencephalopathy, as demonstrated by clinical, virologic, and radiologic response to treatment. However, the treatment also resulted in immune reconstitution inflammatory syndrome and life-threatening immune-related adverse events. These conditions were treated with corticosteroids, leading to treatment resistance.

Progressive multifocal leukoencephalopathy (PML) is a devastating infectious disease of the brain that is caused by JC virus (JCV) in the context of cellular immunodeficiency. To date, no effective antiviral treatment for PML exists, and survival depends on the person’s ability to achieve timely immune reconstitution. Otherwise, the prognosis is particularly grim; the mortality rate is 90% for hematologic malignancy–associated PML (*1*). Immune checkpoints are costimulatory and coinhibitory molecules usually expressed on the surface of immune cells and modulating their activation. Several authors have reported successful PML treatment using immune checkpoint inhibitors (ICIs) targeting programmed cell death protein 1 (PD1), but whether ICIs targeting other proteins such as programmed death-ligand 1 (PD-L) could also treat PML is unknown (*2*).

A 77-year-old woman living in Belgium and with medical history of asymptomatic interstitial lung disease and B-cell chronic lymphocytic leukemia treated with chlorambucil and obinutuzumab was admitted for aphasia, cerebellar ataxia, and cognitive decline that had progressed over 3 months. Complete blood count and flow cytometry revealed lymphopenia affecting all lymphocyte subsets (280 CD4+ cells/μL, 80 CD8+ cells/μL, 30 CD19+ cells/μL). Brain magnetic resonance imaging (MRI) showed T2-weighted hyperintense, nonenhancing, multifocal white matter lesions (Appendix Figure 1). Analysis of cerebrospinal fluid (CSF) revealed 733,845 JCV copies/mL, which enabled a definite diagnosis of PML (*3*). To treat PML, we administered atezolizumab, an anti–PD-L1 humanized monoclonal antibody, at 1,200 mg every 3 weeks. Clinical follow-up consisted of daily physical and neurologic examinations. To monitor immune exhaustion, we performed immunophenotyping on blood specimens by using multicolor flow cytometry the day before and 5 weeks after treatment initiation.

One week after treatment initiation, we noted improvement of aphasia and cognitive function. The next week, the patient experienced abdominal pain, psoriasis-like skin lesions, an episode of transient third-degree atrioventricular block, and a right hemicorporeal clonic seizure, after which mental status was persistently altered. JCV load in the CSF was considerably reduced to 945 copies/mL (Figure). Brain MRI showed progression of lesions visualized on T2 and fluid-attenuated inversion recovery sequences and an increased apparent diffusion coefficient signal, compatible with vasogenic edema (Appendix Figure 1). Despite the absence of classical immune reconstitution inflammatory syndrome (IRIS) features, including gadolinium enhancement, we considered these radiologic characteristics, together with a paradoxical clinical deterioration in viral clearance, to be markers of immune reconstitution. Suspecting IRIS and skin, cardiac, and enteral immune-related adverse events (IRAEs), we administrated intravenous methylprednisolone (1 g/d for 10 d), followed by oral taper over 6 weeks. This regimen resulted in a substantial improvement of her mental status, decrease of the edema seen on brain MRI, and resolution of all other systemic complications. However, 3 weeks after corticosteroid initiation, the patient demonstrated progressive decrease of alertness, new rise of viral load in the CSF, and expansion of PML lesions as shown on brain MRI (Figure). She died of aspiration pneumonia 3 weeks later.

**Figure:**
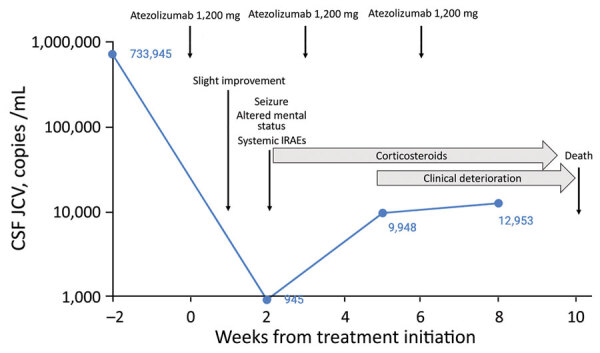
Clinical course and evolution of JC virus load in CSF of 77-year-old patient undergoing atezolizumab therapy for progressive multifocal leukoencephalopathy. CSF, cerebrospinal fluid; IRAEs, immune-related adverse events; JCV, JC virus.

In parallel, atezolizumab treatment was associated with a decrease in detection of PD1 on CD8+ T cells in peripheral blood, but its expression on CD4+ cells remained unchanged (Appendix Figure 2). We observed no substantial change in CD3+, CD4+, and CD8+ cell counts after treatment.

In this case, atezolizumab successfully counteracted immune exhaustion to reinvigorate JCV immunity as reflected by several elements: the initial clinical improvement, the reduction of PD1 expression on blood CD8+ T cells, the marked JCV load reduction in CSF, and the development of a clinical IRIS. However, the clinical IRIS and the severe life-threatening IRAEs required administration of high-dose corticosteroids. Because corticosteroids impair JCV-specific T-cell response and mitigate beneficial ICIs effects (*4*,*5*), methylprednisolone likely resulted in treatment resistance, which led to PML progression and, ultimately, death.

Evidence is growing that immune exhaustion, and notably the PD1 pathway, is involved in PML pathophysiology (*6*). PD1-expressing lymphocytes colocalize with PD-L1+ macrophages in PML lesions, thereby indicating they might function as T-cell partners in immune exhaustion (*7*). Considering the history of interstitial lung disease in our patient, we chose to target PD-L1 to leave intact the interaction between PD1 and its alternative ligand, PD-L2, which had the theoretical benefit of promoting self-tolerance in the lungs, where the PD1/PDL-2 pathway plays a role in regulating inflammation (*8*). Accordingly, despite a striking systemic inflammatory response, our patient did not experience pulmonary IRAE.

Treating PML with ICIs targeting proteins other than PD1 opens the way to a new therapeutic strategy: reinvigorating JCV immunity by using combinations of ICIs. In cancer therapy, compensatory upregulation of alternative immune checkpoints is 1 of the mechanisms of ICI resistance, and PD1/PD-L1 pathway blockade is already combined with inhibition of cytotoxic T lymphocyte antigen 4 to treat metastatic melanoma. Moreover, novel ICIs are being developed, and their combination with current ICIs is already considered a possibility (*9*). Because upregulation of alternative immune checkpoints has been observed in unsuccessful PML treatment with anti-PD1 antibodies (*10*), patients with PML might also benefit from these promising synergic therapeutic combinations.

Appendix Additional information about atezolizumab treatment for progressive multifocal leukoencephalopathy
