# Supplementary material for: Atezolizumab Treatment for Progressive Multifocal Leukoencephalopathy
Source: Emerg Infect Dis. 2022 Jan;28(1):253–6. doi: 10.3201/eid2801.204809 (PMC8714204; doi:10.3201/eid2801.204809)
Supplement: Appendix — Additional information about atezolizumab treatment for progressive multifocal leukoencephalopathy [file 20-4809-Techapp-s1.pdf]

Article DOI: https://doi.org/10.3201/eid2801.204809

Atezolizumab Treatment for Progressive Multifocal Leukoencephalopathy

Appendix

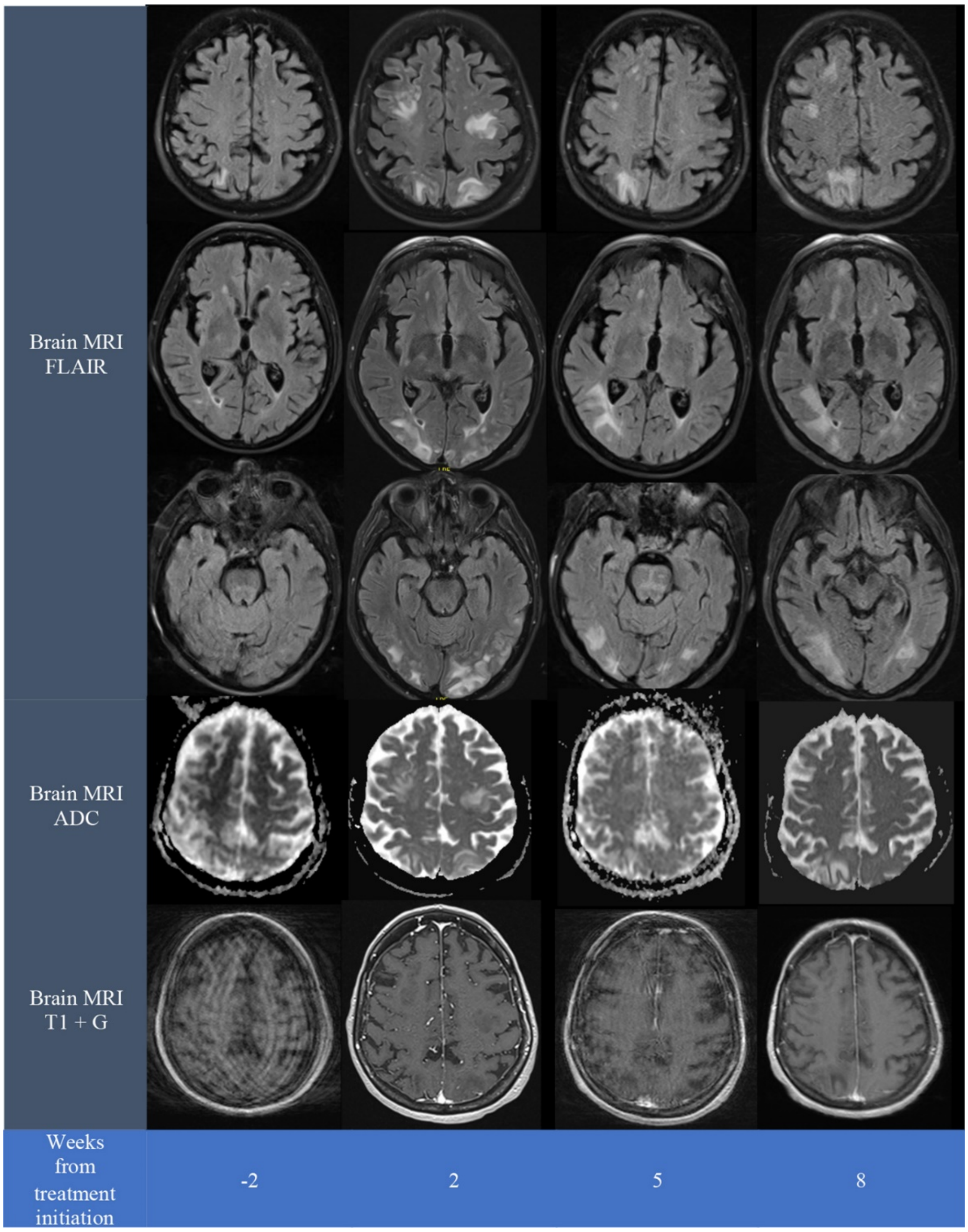

Appendix Figure 1. Evolution of brain MRI scans of a 77-year-old woman undergoing atezolizumab therapy for progressive multifocal leukoencephalopathy. ADC, apparent diffusion coefficient; FLAIR, fluid attenuated inversion recovery; MRI, magnetic resonance imaging; T1 + G, T1 after gadolinium injection.

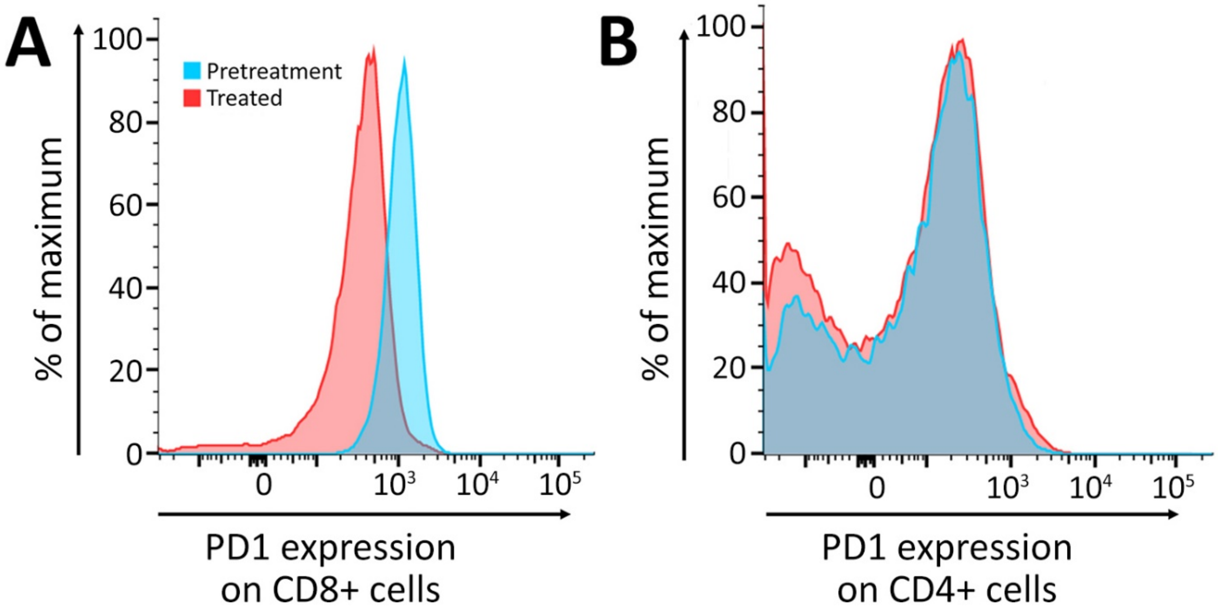

A
100
80
60
40
20
0
% of maximum
Pretreatment
Treated
0
10³
10⁴
10⁵
PD1 expression
on CD8+ cells
B
100
80
60
40
20
0
% of maximum
0
10³
10⁴
10⁵
PD1 expression
on CD4+ cells
